# Synchronous atypical lobular endocervical glandular hyperplasia of the cervix and endometrium: a clinicopathologic case report and review of literature

**DOI:** 10.1186/s13256-026-05906-2

**Published:** 2026-03-05

**Authors:** Ziqi Ge, Xiaoping Wang, Zili Zhang, Junmin Li, Chen Jiang, Tian Luo

**Affiliations:** 1 Department of Gynecology, Jinan Maternity and Child Care Hospital, Jinan, 250001 Shandong China; 2 Department of Pathology, Jinan Maternity and Child Care Hospital, Jinan, 250001 Shandong China; 3 Department of General Surgery, Jinan Fourth People’s Hospital, Jinan, 250031 Shandong China

**Keywords:** Atypical lobular endocervical glandular hyperplasia, Gastric-type endocervical adenocarcinoma, Hysteroscopy, Synchronous mucinous metaplasia and neoplasia of the female genital tract

## Abstract

**Background:**

Atypical lobular endocervical glandular hyperplasia is an infrequent precursor to gastric-type cervical adenocarcinoma, often posing diagnostic challenges owing to its occult nature and high misdiagnosis rate. The synchronous presentation in both the cervix and endometrium, a form of synchronous mucinous metaplasia and neoplasia of the female genital tract, is even rarer.

**Case presentation:**

We report the case of a 53-year-old Chinese woman with increased vaginal discharge and postmenopausal bleeding, initially misdiagnosed as cervical and endometrial polyps. Postoperative pathology following hysteroscopy revealed synchronous atypical lobular endocervical glandular hyperplasia of the cervix and endometrium. Notably, while atypical lobular endocervical glandular hyperplasia is typically an occult finding diagnosed after cervical conization, hysteroscopy in this case enabled direct visualization and targeted biopsy, overcoming a significant diagnostic hurdle.

**Conclusion:**

This case underscores the insidious clinical presentation of synchronous atypical lobular endocervical glandular hyperplasia and highlights the pivotal role of hysteroscopy as a diagnostic tool. It can facilitate the early and accurate identification of these occult lesions, thereby preventing potential misdiagnosis and guiding appropriate management.

## Background

Lobular endocervical glandular hyperplasia (LEGH) is a benign proliferative lesion of the cervical glands. Pathological observation revealed larger glandular structures in the center of the lobule surrounded by densely distributed smaller- to medium-sized glands. The glands are covered by tall columnar mucinous epithelium with eosinophilic granular cytoplasm and basal nuclei [1]. Atypical lobular endocervical glandular hyperplasia (ALEGH) demonstrates cytological and architectural atypia in addition to LEGH but lacks stromal infiltration and exhibits features of gastric differentiation. The atypia is confined to the glandular areas of lobular hyperplasia [2]. ALEGH presents insidiously with atypical symptoms, often accompanied by copious mucus or watery vaginal discharge [3]. Human papilloma virus (HPV) screening is often negative. This insidious nature contributes to a high risk of misdiagnosis and potential disease progression. The synchronous occurrence of these mucinous lesions in both the cervix and endometrium, a rare manifestation of synchronous mucinous metaplasia and neoplasia of the female genital tract (SMMN-FGT), further complicates the diagnostic landscape.

## Case presentation

A 53-year-old Chinese female presented to our hospital with a chief complaint of a 2-year history of increased vaginal discharge and two episodes of postmenopausal bleeding, which began 1 year after menopause. The patient had a 3-year history of hypertension, with irregular medication use, and her blood pressure was maintained at 140–150/70–80 mmHg. She had previously undergone one cesarean section. For contraception, she had used an intrauterine device (IUD) for 20 years, which was removed 1 year prior to presentation. Gynecological examination revealed a smooth cervix with a polypoid growth at the external os, which did not bleed on contact. Both adnexal areas were slightly thickened and nontender, with no rebound tenderness. Laboratory and imaging studies revealed the following: fasting blood glucose 6.7 mmol/L (3.9–6.1 mmol/L), glycated hemoglobin 5.8% (4–6%), triglycerides 3.78 mmol/L (0.56–1.69 mmol/L), total cholesterol 6.44 mmol/L (2.86–5.98 mmol/L), and low-density lipoprotein cholesterol 4.55 mmol/L (2–4.13 mmol/L); cervical HPV was negative; and liquid-based cytology test (ThinPrep Cytologic Test, TCT) indicated atypical glandular cells (AGC). Hormonal assessment revealed an elevated estradiol level of 90.95 pg/mL (postmenopausal reference [ref]: < 20–40 pg/mL) and unexpectedly low levels of follicle-stimulating hormone (FSH) at 8.78 mIU/mL (ref: 16.74–113.59 mIU/mL) and luteinizing hormone (LH) at 3.55 mIU/mL (ref: 10.87–58.64 mIU/mL) for her postmenopausal status. Other hormone levels, including progesterone (0.18 ng/mL; ref: 0.08–0.78 ng/mL), prolactin (9.77 ng/mL; ref: 2.74–19.64 ng/mL), and testosterone (30.51 ng/dL; ref: 10–75 ng/dL), were within their respective normal ranges. All tumor markers (alpha-fetoprotein, carbohydrate antigen 125, CA199, carcinoembryonic antigen, CA153, human epididymis protein 4, and squamous cell carcinoma antigen) were negative. Gynecological ultrasound showed endometrial thickness about 8 mm, uterine leiomyoma, a strip-like echogenic lesion measuring 27 mm × 7 mm within the cervical canal suggestive of polyp; left adnexal region demonstrated a tubular anechoic area, suggesting hydrosalpinx; and a right ovarian cyst categorized as Ovarian-Adnexal Reporting and Data System (O-RADS) category 2.

Firstly, the patient underwent hysteroscopic cervical polypectomy, endometrial lesion resection, and fractional diagnostic curettage. Postoperative pathological examination revealed the following: Postoperative pathology of the cervical specimen revealed a cervical polyp with LEGH in the overlying glands, showing focal features of ALEGH, and chronic inflammation. The endometrial polyp specimen also showed LEGH with at least focal areas of ALEGH. Separately, the fragmented endometrium from diagnostic curettage revealed simple endometrial hyperplasia without atypia. Immunohistochemistry revealed the following: MUC6 (+), Ki-67 (approximately 3% positive), p53 (wild-type pattern), HIK1083 (partially positive), and positive Alcian blue-periodic acid–Schiff (AB-PAS) staining (purplish-red). Following hysteroscopic surgery, further pelvic magnetic resonance imaging (MRI) indicated: the bilateral adnexal regions presented tubular structures with high signal intensity on both T1- and T2-weighted images, with focal luminal dilation and septation. The uterus showed retroverted position with mildly inhomogeneous myometrial signals. No endometrial thickening was observed; the mid uterine cavity displayed heterogeneous signals with iso-T2 intensity. The lower uterine cavity and endocervical canal demonstrated heterogeneous signaling with cystic structures showing high signal intensity on both T1- and T2-weighted images. No significant fluid with long T1 or long T2 signals was noted in the pelvic cavity nor was there any obvious pelvic lymphadenopathy. The patient underwent diagnostic cervical conization and total laparoscopic hysterectomy with bilateral salpingo-oophorectomy. Intraoperative frozen section analysis of the cervical tissue revealed a small number of glands with gastric-type differentiation, with focal areas interpreted as ALEGH. Routine postoperative pathology showed: lobular endocervical glandular hyperplasia at the internal cervical os, with focal atypical lobular endocervical glandular hyperplasia in some glands. There was proliferative endometrium and one uterine leiomyoma. A simple serous cyst was present in the left ovary; a luteinized follicular cyst in the right ovary; and chronic salpingitis with hydrosalpinx in the left fallopian tube. Immunohistochemistry revealed MUC6 (partly positive), esterogen receptor (ER, focally positive), and AB-PAS (positive).

## Discussion

The spectrum of gastric-type glandular lesions of the uterine cervix ranges from benign entities to frankly malignant adenocarcinomas. Benign lesions include simple gastric-type mucinous metaplasia (SGM), tunnel clusters (type A), and lobular endocervical glandular hyperplasia (LEGH) [4]. LEGH, believed to arise from pyloric gland metaplasia, is generally considered benign but carries a reported malignant transformation rate of 1.4% [5].

According to the 2018 International Endocervical Adenocarcinoma Criteria and Classification (IECC), both atypical LEGH (ALEGH) and gastric-type adenocarcinoma *in situ* (gAIS) are recognized as precursor lesions of gastric-type endocervical adenocarcinoma (G-EAC). Minimal deviation adenocarcinoma (MDA) is a well-differentiated gastric-type adenocarcinoma, frequently characterized by “claw-shaped” glands of variable size and shape infiltrating the cervical wall. Cytological atypia and stromal response are generally mild; however, the depth of invasion often exceeds the normal distribution range of cervical glands. The close association between glands and thick-walled large blood vessels is a more significant diagnostic clue [6]. The direct link between ALEGH and invasive cancer is supported by studies like that of Talia KL *et al*. [7], who demonstrated that ALEGH was frequently found adjacent to or intermixed with MDA, reinforcing its role as a precursor.

Cervical cytology assists in distinguishing between lobular endocervical glandular hyperplasia (LEGH) and minimal deviation adenocarcinoma/gastric-type adenocarcinoma/atypical LEGH (MDA/GAS/ALEGH). A cytological finding of atypical glandular cells—not otherwise specified (AGC-NOS) typically suggests LEGH, whereas atypical glandular cells—favor neoplastic (AGC-FN) usually indicates ALEGH or MDA/GAS [5, 8]. This ambiguity, coupled with the fact that these lesions are typically HPV negative, complicates early detection. The pathogenesis of ALEGH itself remains unclear, though molecular studies have identified chromosomal abnormalities such as 3q gain and 1p loss, which are absent in nonatypical LEGH, suggesting a neoplastic evolution [9]. Notably, our patient presented with hypertension and dyslipidemia; while an association between metabolic syndrome and ALEGH has not been established, it represents an area for future investigation.

Magnetic resonance imaging (MRI) is a valuable tool for evaluating these lesions, demonstrating high diagnostic accuracy for both LEGH (85–90%) and MDA/GAS (67–70%) [10]. On T2-weighted images, LEGH classically presents with a “cosmos pattern”—a multicystic mass with small cysts surrounding larger central ones—which helps differentiate it from the solid, infiltrative components of adenocarcinoma [11, 12]. An alternative “raspberry pattern,” consisting of tightly clustered small cysts, has also been described, particularly in postmenopausal patients with associated adenocarcinoma in situ (AIS) [13]. In our case, the MRI findings of heterogeneous signals and multicystic structures in the lower uterus and cervix were consistent with these described patterns, though not classic.

Definitive diagnosis, however, relies on histopathological and immunohistochemical examination. The cornerstone of these lesions is their gastric-type differentiation, identifiable by neutral mucins that stain rose-red with periodic acid–Schiff (PAS) and deep blue with Alcian blue, a feature clearly observed in our patient [9]. Histologically, LEGH is defined by a lobular proliferation of benign-appearing glands with pale, mucin-rich cytoplasm and basal nuclei, without stromal invasion [1]. ALEGH is distinguished by the addition of specific atypical features, including nuclear enlargement, irregular nuclear membranes, prominent nucleoli, and loss of polarity [4].

Immunohistochemistry (IHC) is crucial for refining the diagnosis and assessing malignant potential. While both LEGH and ALEGH typically express gastric-type markers like MUC6 and HIK1083, their expression patterns and proliferation markers differ. For instance, the Ki-67 proliferation index is generally low in LEGH (< 10%) but can be elevated in ALEGH (0.7–35.7%) [14]. Although our patient’s Ki-67 index was low (~3%), the diagnosis of ALEGH was supported by distinct cytological atypia and strong MUC6 positivity. Furthermore, p53 status can be informative; both LEGH and ALEGH usually show a wild-type p53 pattern, whereas over 50% of advanced GAS cases exhibit a mutant-type pattern [14, 15]. The expression of HIK1083 may be higher in LEGH than in its atypical or malignant counterparts [16], and TFF2 has been proposed as another useful marker for gastric phenotype [17]. The constellation of pathological findings in our case—focal atypia, positive MUC6 and AB-PAS, and wild-type p53—was fully consistent with the diagnosis of ALEGH.

**Fig. 1:**
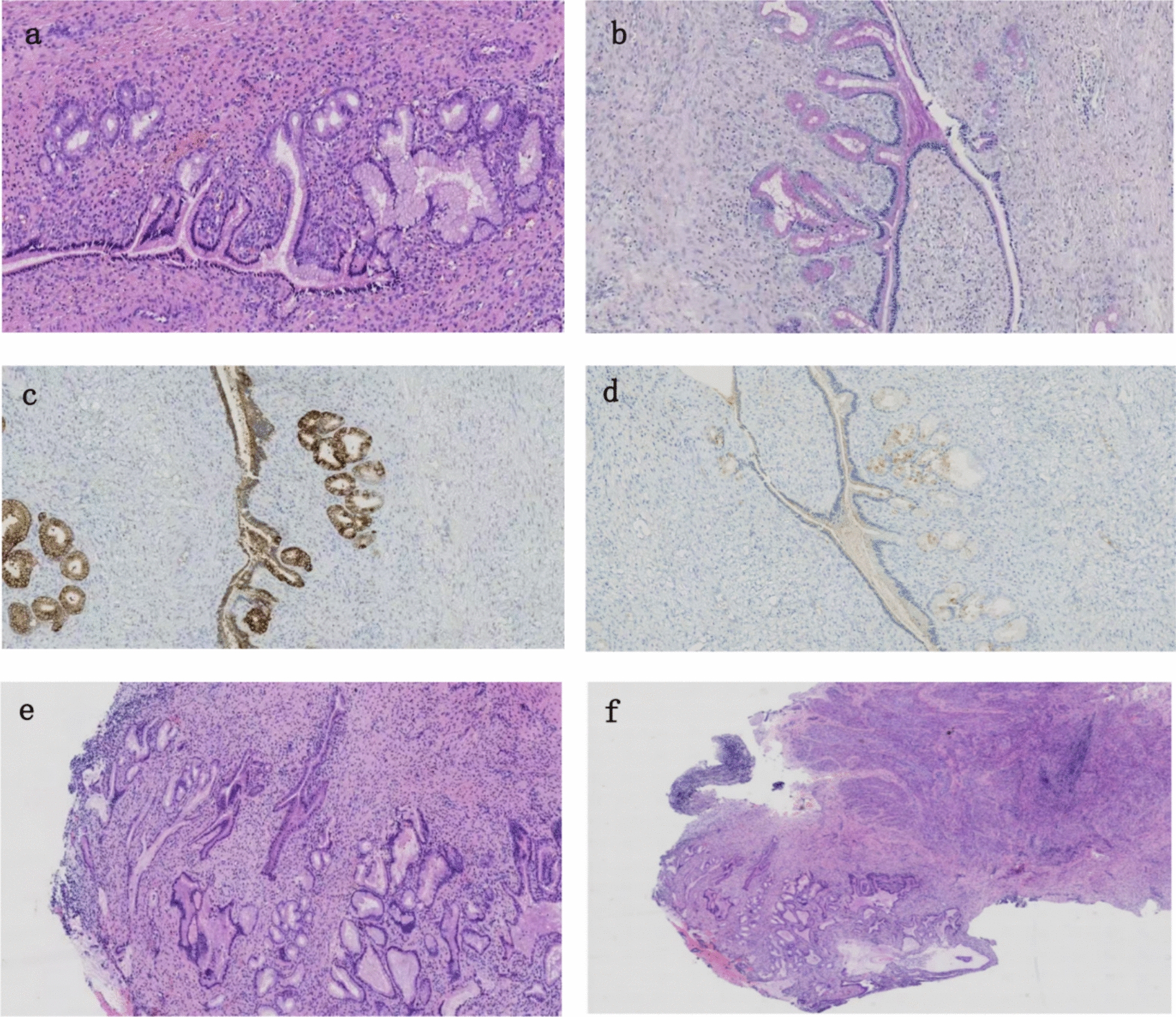
Histopathological and immunohistochemical findings. **a, b** Hematoxylin–eosin staining of the cervical lesion showing lobular endocervical glandular hyperplasia characterized by densely packed small-to-medium glands surrounding larger central ducts, forming a lobular architecture. Focal areas exhibit cytological atypia, including nuclear hyperchromasia and eosinophilic cytoplasm, consistent with atypical lobular endocervical glandular hyperplasia. **c** Immunohistochemical staining of the cervical lesion shows strong positivity for MUC6. **d** The cervical lesion is weakly positive for HIK1083. **e, f** Hematoxylin–eosin staining of the endometrial lesion, located in the lower uterine segment, also demonstrates features of atypical lobular endocervical glandular hyperplasia, with hyperchromatic nuclei and eosinophilic cytoplasm. The lobular structure is less distinct in some areas. A small portion of normal endometrium is visible in the upper-left corner of panel (**e**)

The pathological diagnosis for this patient is consistent with ALEGH (Fig. 1).

Synchronous mucinous metaplasia and neoplasia of the female genital tract (SMMN-FGT) manifests as mucinous lesions simultaneously present in two or more regions of the female reproductive tract (cervix, uterus, fallopian tubes, ovaries, or peritoneum), and includes a spectrum of gastric-type differentiated non-neoplastic, benign, borderline, and malignant lesions. Morphological features encompass simple gastric-type mucinous metaplasia, papillary growth, atypical papillary growth, microinvasive adenocarcinoma, gastric-type adenocarcinoma, tubal mucinous metaplasia, ovarian mucinous cystadenoma, and borderline or mucinous ovarian carcinoma. Kuragaki *et al*. found that STK11 gene mutations may contribute to SMMN-FGT associated with lobular endocervical glandular hyperplasia/preneoplastic gastric-type metaplasia (LEGH/PGM) [18]. Studies indicate that GNAS gene mutations may play a role in the malignant transformation of lobular lesions [19]. Lu Linghui *et al*. reported 25 cases of SMMN-FGT, with patient ages ranging from 33 to 75 years (median 46 years), and 8 cases involving both the cervix and corpus uteri; immunohistochemistry revealed variable positive expression of MUC6, diffuse and strong expression of CK7 in all cases, and marked heterogeneity in Ki-67 proliferation index (1–60%) [20]. Mikami *et al*. described four LEGH patients with lesions affecting both cervix and endometrium, in which all mucinous lesions were positive for HIK1083 and/or MUC6 markers. It is postulated that LEGH/PGM may occur in a continuous or multifocal manner in the female reproductive tract, originating from Müllerian duct-derived epithelium, and may subsequently lead to borderline mucinous tumors and/or adenocarcinoma. In certain instances, distinguishing whether the lesions are synchronous independent mucinous lesions or metastatic disease from one site to another may be challenging [21]. In our patient, the cervical lesion was located on the left posterior wall of the internal os, whereas the uterine lesion was on the right lateral wall of the lower uterine segment. These distinct locations support the diagnosis of two independent, synchronous ALEGH sites rather than a single lesion with contiguous extension or metastasis.

Traditionally, cervical conization has been the standard method for obtaining tissue from cervical lesions [22]. However, for lesions like ALEGH, which are typically located high in the endocervical canal near the internal os, conization presents significant limitations [6]. Achieving an adequate sample from this high location can be challenging, potentially leading to missed diagnoses. Furthermore, deep conization carries substantial risks, including future cervical incompetence and stenosis, which can impair fertility in women of reproductive age.

In this context, hysteroscopy emerges as a superior diagnostic tool, a point strongly underscored by our case. Given the nonspecific symptoms and negative HPV screening typical of ALEGH, a direct visual approach is invaluable. Hysteroscopy allows for precise visualization of the lesion’s location, morphology, and extent, facilitating targeted biopsies or complete excision under direct sight. This targeted approach not only maximizes diagnostic accuracy but also minimizes iatrogenic damage to the cervix.

The diagnostic advantage of hysteroscopy is supported by emerging evidence. A study by Shiro *et al*. directly compared hysteroscopic biopsy with conization for suspected LEGH-related lesions. They reported a dramatically higher sensitivity for hysteroscopy (100%) compared with conization (a mere 6%), with a perfect negative predictive value (100%) [23]. Our case serves as a real-world validation of these findings: hysteroscopy not only identified the cervical polyp but also revealed the synchronous endometrial lesion, enabling a complete and accurate diagnosis that might have been missed or delayed with conventional methods alone.

**Fig. 2:**
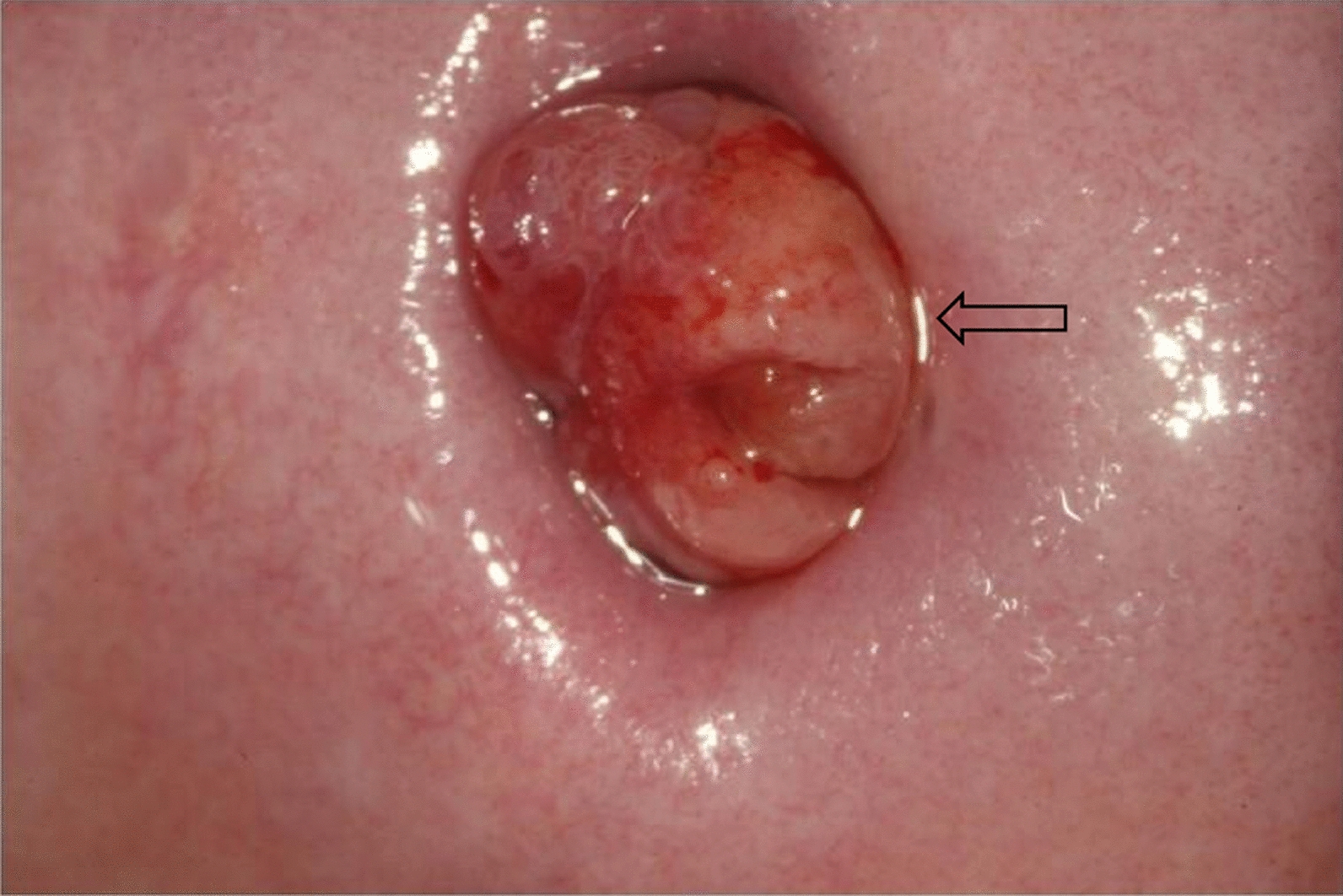
The arrow indicates cervical polyps. Colposcopic view of the cervix. A polypoid lesion with a smooth surface and prominent vascularity is visible at the external os. No contact bleeding was observed

**Fig. 3:**
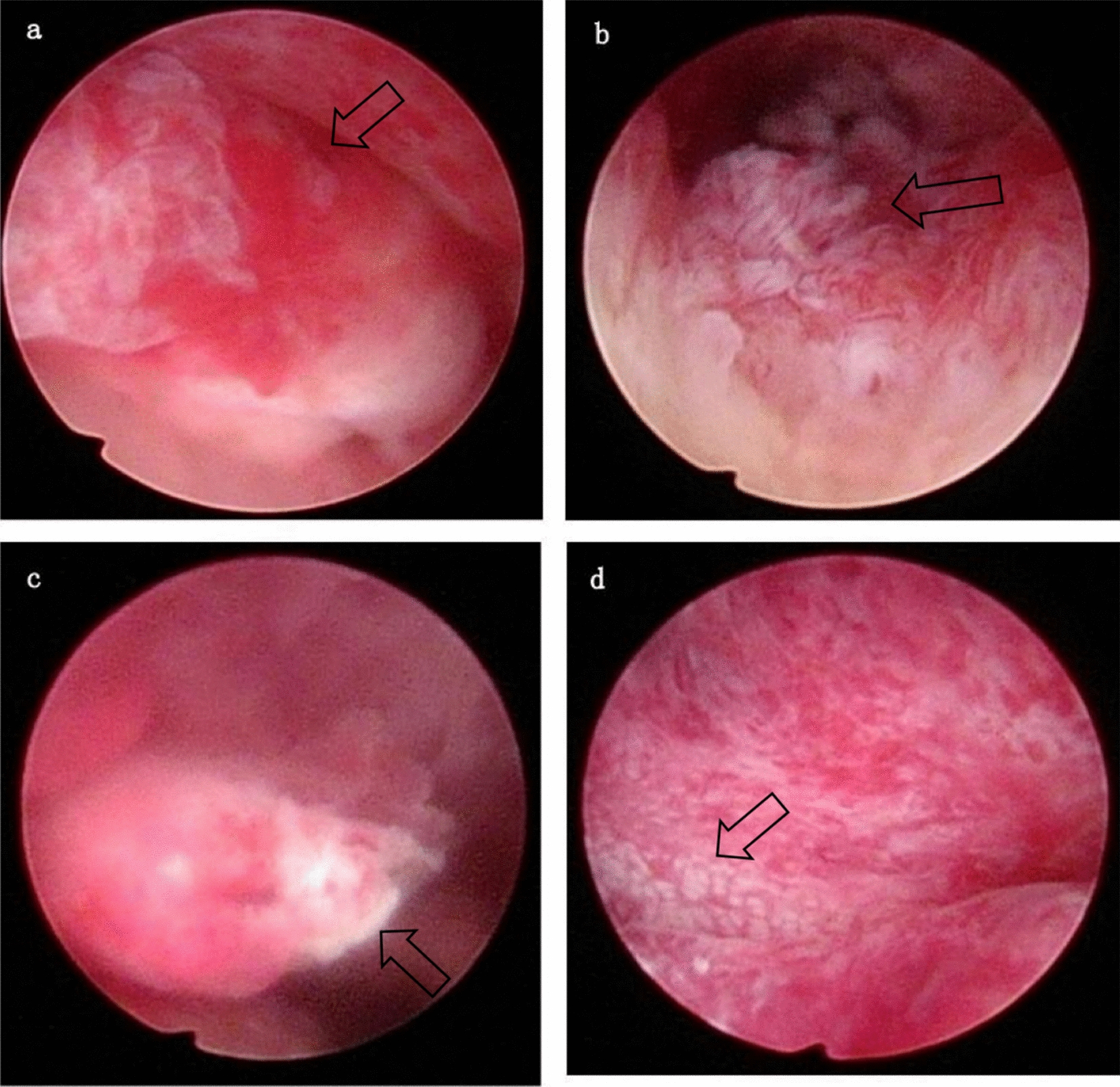
The arrows indicate the following, (**a**) A polypoid lesion with a smooth surface and rich vascularity is seen in the endocervical canal. **b** The richly vascularized pedicle of the cervical polyp after resection. **c** A smaller, finger-like polypoid growth is visible on the right wall of the mid-uterine cavity. **d** The endometrium appears unevenly thickened with a hilly surface and a lattice-like vascular pattern at the fundus

Colposcopic images are shown in Fig. 2 and hysteroscopic images in Fig. 3.

Total hysterectomy is generally considered the appropriate treatment for ALEGH. Synchronous mucinous metastatic neoplasm and female genital tract tumors (SMMN-FGT) are exceedingly rare and generally have a relatively favorable prognosis. In young patients or those with fertility concerns, lesion excision may be performed, but negative surgical margins must be confirmed pathologically.

## Conclusion

We have reported a rare case of synchronous ALEGH of the cervix and endometrium, a form of SMMN-FGT. This case highlights the insidious nature of ALEGH and underscores the high diagnostic value of hysteroscopy, particularly in patients with nonspecific symptoms such as increased vaginal discharge, for achieving an early and accurate diagnosis.

## Data Availability

All data are included within the article.

## Abbreviations

LEGH: Lobular endocervical glandular hyperplasia
ALEGH: Atypical lobular endocervical glandular hyperplasia
HPV: Human papilloma virus
AGC: Atypical glandular cells
gAIS: Gastric-type adenocarcinoma in situ
G-EAC: Gastric-type endocervical adenocarcinoma
MDA: Minimal deviation adenocarcinoma
AGC-NOS: Atypical glandular cells—not otherwise specified
AGC-FN: Atypical glandular cells—favor neoplasia
SMMN-FGT: Synchronous mucinous metaplasia and neoplasia of the female genital tract
SGM: Simple gastric metaplasia
PGM: Pyloric gland metaplasia
PAS: Periodic acid–Schiff
HE: Hematoxylin‒eosin staining
TFF2: Trefoil factor family 2 protein

## References

[1] Mikami Y, Hata S, Melamed J, Fujiwara K, Manabe T. Lobular endocervical glandular hyperplasia is a metaplastic process with a pyloric gland phenotype. Histopathology. 2001;39(4):364–72. 10.1046/j.1365-2559.2001.01239.x. 11683936 10.1046/j.1365-2559.2001.01239.x

[2] Zhang J. New progress in pathological diagnosis of cervical adenocarcinoma and its precancerous lesions. Chin J Obstet Gynecol. 2022;57(10):785–8.

[3] Nucci MR, Clement PB, Young RT. Lobular endocervical glandular hyperplasia, not otherwise specified. Am J Surg Pathol. 1999;23:886–91. 10.1097/00000478-199908000-00005. 10435557 10.1097/00000478-199908000-00005

[4] Mikami Y, Kiyokawa T, Hata S, Fujiwara K, Moriya T, Sasano H, *et al*. Gastrointestinal immunophenotype in adenocarcinomas of the uterine cervix and related glandular lesions: a possible link between lobular endocervical glandular hyperplasia/pyloric gland metaplasia and “adenoma malignum.” Mod Pathol. 2004;17:962–72. 10.1038/modpathol.3800148. 15143335 10.1038/modpathol.3800148

[5] Kobara H, Miyamoto T, Ando H, *et al*. Limited frequency of malignant change in lobular endocervical glandular hyperplasia. Int J Gynecol Cancer. 2020;30(10):1480–7. 10.1136/ijgc-2020-001612. 32883699 10.1136/ijgc-2020-001612 PMC7548537

[6] Stolnicu S, Talia KL, McCluggage WG. The evolving spectrum of precursor lesions of cervical adenocarcinomas. Adv Anat Pathol. 2020;27:278–93. 10.1097/PAP.0000000000000266. 32482967 10.1097/PAP.0000000000000266

[7] Talia KL, Stewart C, Howitt BE, *et al*. HPV-negative gastric type adenocarcinoma in situ of the cervix: a spectrum of rare lesions exhibiting gastric and intestinal differentiation. Am J Surg Pathol. 2017;41(8):1023–33. 10.1097/PAS.0000000000000855. 28394803 10.1097/PAS.0000000000000855

[8] Kobara H, Miyamoto T, Otsuki T, Ohya A, Shiozawa T. Worsening cytology and lesion enlargement are useful indicators for malignant transformation of lobular endocervical glandular hyperplasia during follow-up: a case report. Gynecol Oncol Rep. 2020;32: 100571. 10.1016/j.gore.2020.100571. 32373692 10.1016/j.gore.2020.100571 PMC7191578

[9] Kawauchi S, Kusuda T, Liu XP, *et al*. Is lobular endocervical glandular hyperplasia a cancerous precursor of minimal deviation adenocarcinoma?: a comparative molecular-genetic and immunohistochemical study. Am J Surg Pathol. 2008;32(12):1807–15. 10.1097/PAS.0b013e3181883722. 18779726 10.1097/PAS.0b013e3181883722

[10] Selenica P, Alemar B, Matrai C, Talia KL, Veras E, Hussein Y, *et al*. Massively parallel sequencing analysis of 68 gastric-type cervical adenocarcinomas reveals mutations in cell cycle-related genes and potentially targetable mutations. Mod Pathol. 2021;34:1213–25. 10.1038/s41379-020-00726-1. 33318584 10.1038/s41379-020-00726-1 PMC8154628

[11] Takatsu A, Shiozawa T, Miyamoto T, *et al*. Preoperative differential diagnosis of minimal deviation adenocarcinoma and lobular endocervical glandular hyperplasia of the uterine cervix: a multicenter study of clinicopathology and magnetic resonance imaging findings. Int J Gynecol Cancer. 2011;21(7):1287–96. 10.1097/IGC.0b013e31821f746c. 21685796 10.1097/IGC.0b013e31821f746c

[12] Ohya A, Asaka S, Fujinaga Y, Kadoya M. Uterine cervical adenocarcinoma associated with lobular endocervical glandular hyperplasia: radiologic-pathologic correlation. J Obstet Gynaecol Res. 2018;44(2):312–22. 10.1111/jog.13528. 29144012 10.1111/jog.13528

[13] Omori M, Kondo T, Tagaya H, Watanabe Y, Fukasawa H, Kawai M, *et al*. Utility of imaging modalities for predicting carcinogenesis in lobular endocervical glandular hyperplasia. PLoS ONE. 2019;14: e0221088. 10.1371/journal.pone.0221088. 31415639 10.1371/journal.pone.0221088 PMC6695122

[14] Mikami Y, Kojima A, Kiyokawa T, *et al*. Ki67 labelling index and p53 status indicate neoplastic nature of atypical lobular endocervical glandular hyperplasia (ALEGH). Histopathology. 2009;55(3):362–4. 10.1111/j.1365-2559.2009.03346.x. 19723155 10.1111/j.1365-2559.2009.03346.x

[15] Ishii S, Ito T, Yamada M, *et al*. Characteristic cytological findings of lobular endocervical glandular hyperplasia associated with adenocarcinoma of the uterine cervix. Acta Cytol. 2020;64(6):556–62. 10.1159/000509667. 32814324 10.1159/000509667

[16] Yamanoi K, Ishii K, Tsukamoto M, Asaka S, Nakayama J. Gastric gland mucin-specific O-glycan expression decreases as tumor cells progress from lobular endocervical gland hyperplasia to cervical mucinous carcinoma, gastric type. Virchows Arch. 2018;473(3):305–11. 10.1007/s00428-018-2381-6. 29845361 10.1007/s00428-018-2381-6

[17] Asaka S, Nakajima T, Momose M, Miyamoto T, Uehara T, Ota H. Trefoil factor family 2 protein: a potential immunohistochemical marker for aiding diagnosis of lobular endocervical glandular hyperplasia and gastric-type adenocarcinoma of the uterine cervix. Virchows Arch. 2019;474(1):79–86. 10.1007/s00428-018-2469-z. 30324235 10.1007/s00428-018-2469-z

[18] Kuragaki C, Enomoto T, Ueno Y, *et al*. Mutations in the STK11 gene characterize minimal deviation adenocarcinoma of the uterine cervix. Lab Invest. 2003;83(1):35–45. 10.1097/01.lab.0000049821.16698.d0. 12533684 10.1097/01.lab.0000049821.16698.d0

[19] Ando H, Miyamoto T, Kashima H, *et al*. Usefulness of a management protocol for patients with cervical multicystic lesions: a retrospective analysis of 94 cases and the significance of GNAS mutation. J Obstet Gynaecol Res. 2016;42(11):1588–98. 10.1111/jog.13083. 27718288 10.1111/jog.13083 PMC5108490

[20] Linghui L, Yiqing C, Jing L, *et al*. Clinical and pathological characteristics of mucinous metaplasia and tumors in multiple sites of the female reproductive tract at the same time. Chinese J Oncol. 2024;46(12):1195–208. 10.3760/cma.j.cn112152-20240518-00201.

[21] Mikami Y, Kiyokawa T, Sasajima Y, *et al*. Reappraisal of synchronous and multifocal mucinous lesions of the female genital tract: a close association with gastric metaplasia. Histopathology. 2009;54(2):184–91. 10.1111/j.1365-2559.2008.03202.x. 19207943 10.1111/j.1365-2559.2008.03202.x

[22] Miyamoto T, Kobara H, Shiozawa T. Biology and management of lobular endocervical glandular hyperplasia. J Obstet Gynaecol Res. 2022;48(12):3056–67. 10.1111/jog.15441. 36177810 10.1111/jog.15441 PMC10092153

[23] Shiro R, Kotani Y, Ohta M, *et al*. Diagnostic utility of hysteroscopic biopsy in suspected lobular endocervical glandular hyperplasia. Healthcare. 2023;11(11):1619. 10.3390/healthcare11111619. 37297759 10.3390/healthcare11111619 PMC10252663

